# Antecedents and Consequences of Green Mindfulness: A Conceptual Model

**DOI:** 10.3390/ijerph19116367

**Published:** 2022-05-24

**Authors:** Yi-Hui Ho, Cheng-Kun Wang, Chieh-Yu Lin

**Affiliations:** 1 Department of International Business, Chang Jung Christian University, Tainan 71101, Taiwan; vicky@mail.cjcu.edu.tw; 2 College of Management, Chang Jung Christian University, Tainan 71101, Taiwan; brian19.brian19@gmail.com

**Keywords:** green mindfulness, environmental management, antecedents, consequences, multilevel model

## Abstract

While many companies take the environmental environment as a fundamental part of their business strategies, managers are facing the challenges to explore the integration of environmental concepts and business operations. Although there are an amount of studies about environmental management in the literature, only a few of them applied the concept of mindfulness to environmental management. Mindfulness is regarded as a way of operation marked by the willingness to consider alternative perspectives, focus on the present, attention to operational detail, and interest in exploring and understanding failures. This study suggests that companies require keeping mindfulness in environmental management implementation. Therefore, this paper aims to explore the application of mindfulness theory to environmental management, and propose a conceptual model of antecedents and consequences of green mindfulness. The proposed multilevel model describes the influences of organizational and individual antecedents on green mindfulness, and the organizational and individual consequences of green mindfulness.

## 1. Introduction

In the context of growing global concerns on environmental issues, an increasing number of firms are attentive to enhancing their competitiveness through reducing the impact of environment on the manufacturing and service activities, addressing the environmental concerns of their customers, and achieving improvements in the environmental performance. Many firms regard commitment to the environment as an important variable within the current competitive scenarios, and engage in environmental management in reaction to the rise in environmental legislation and public concern about environmental degradation [1,2]. Environmental management has received sustained research interest over time due to the multitude of factors that can affect environmental efforts. Many researchers have used a variety of theories, including institutional theory, resource-based view, stakeholder theory, innovation diffusion theory and others, to propose various explanations for firms’ implementation of environmental management.

Engaging in environmental management is usually believed to be able to confer competitive benefits to firms. Firms can achieve considerable environmental performance by successfully implementing green practices in their work systems [3,4]. However, these practices might not attain the thorough usage beyond the first adoption because engaging in environmental management often constitutes complex technologies and processes, and calls for significant investment of organizational resources [3,5,6]. Winn and Angell [7] addressed that corporate greening may begin with top management’s awareness of the need for responses to environmental issues, and may ideally end with successful implementation in the company. Successful implementation of environmental management relies on full engagement when evolving operating responses to issues on the environment.

Implementing environmental management embraces complex information processing and decision-making which includes making sense of innovative green concepts or practices which a company is unacquainted with, and is normally considered as being uncertain and ambiguous over the implementing process outcomes [7,8,9]. Accordingly, supervisors encountered the duty of analyzing the ramifications of the green concepts or practices of their companies [10]. In the circumstances, deciding on whether a specific green practice is beneficial for the firm, whether the execution timing is appropriate, and how the implementation can be conducted well, requires companies to be mindful of engaging in environmental management with reasoning grounded in their facts and specifics [6,11,12]. In organizational decision-making, mindfulness means a state of being alert and aware. According to the resource-based view, mindfulness can be regarded as an important resource to reinforce competitive advantage [13,14]. It is this disposition that is believed to support in making contextually differentiated interpretations of situations and information scenarios [15,16,17].

While applying environmental criteria into business operations requires exploring the combination of new resources and deploying existing resources in new ways [18,19], implementing environmental management can be regarded as an organizational innovation process [20,21,22,23]. In an attempt to understand how a firm implements new technologies or processes, the notion of mindfulness has been introduced to investigate differences in innovative behavior among organizations [24,25,26]. In the context of organizational innovation implementations, mindfulness corresponds to an engagement with an innovation based on the facts that are unique to the firm [6,26,27]. Mindfulness will reduce the possibility of failure when applying innovations because mindfulness can bring about the decisions based on contextually relevant understandings of a given condition [25,26]. As mindfulness is desirable in innovation implementation processes, firms also require mindfulness thinking in the implementation of environmental management.

The approach of mindfulness identifies the management flexibility values in structuring and timing investment decisions while encountering unclear circumstances, varying the risks of the investment proposal at different stages [15,24,28]. Accordingly, the theory of mindfulness is suitable for the application to investigating firms’ environmental management employment. Firms must keep mindfulness thinking once engaging in environmental management [10]. Therefore, it is essential to understand the issues of mindfulness in implementing environmental management within firms.

According to a review on the literature related to mindfulness theory and environmental management, the notion of mindfulness has been less used in the research of environmental management [6,28,29], although the mindfulness concept has been deliberated in numbers of studies across diverse disciplines or subjects [30,31,32,33]. To fill the research gap, this paper considers that it is necessary to keep mindfulness in implementing environmental management within firms, and proposes the concept of green mindfulness.

This paper attempts to explore the application of the mindfulness concept to environmental management and build a conceptual framework describing antecedents and consequences of green mindfulness. Understanding predictors of green mindfulness can help firms appreciate the potential drivers and barriers to implementing environmental management. Analyzing effects of green mindfulness can help firms learn about the influences of green mindfulness on firms’ performance.

Mindfulness has been introduced into organizational studies through the investigation of individual and organizational mindfulness within an organization. Organizational mindfulness is the connection and sharing of the mindfulness of individuals to help both individuals and the organization achieve greater congruence between their intentions and outcomes [16,34]. Therefore, the green mindfulness in this study is divided into individual green mindfulness and organizational green mindfulness. Both antecedents and consequences of green mindfulness are also analyzed based on individual and organizational levels. Recognizing the organizational mindfulness phenomena being inherently multilevel in nature [6,35], analyzing the antecedents and consequences of green mindfulness should be from a multilevel perspective.

Firms provide important organizational contexts for influencing individuals’ behaviors, which includes the attributes and processes that facilitate or constrain green mindfulness. Implementing environmental management usually requires a great deal of individual effort and persistence [20,28]. However, less attention has been given to understanding potential antecedents and consequences of mindfulness [6,35]. Regarding environmental management, many studies have discussed the influences of a variety of organizational and individual factors on environmental strategy, and the organizational and individual consequences of environmental management. Therefore, a multilevel conceptual model of green mindfulness is proposed in this study. Drawing on the resource-based view and a review of related literature, we propose five organizational antecedents and three individual antecedents that may be particularly important in light of green mindfulness. The influences of green mindfulness on organizational and individual outcome will also be discussed.

In summary, this study aims to apply the concept of mindfulness to environmental management and propose a multilevel conceptual model describing the influences of organizational and individual antecedents on green mindfulness, and the organizational and individual consequences of green mindfulness. The recognition of the key role of green mindfulness in firms’ environmental management would be valuable for both managerial and research purposes. Understanding antecedents and consequences of green mindfulness is imperative for implementing environmental management and for investigators to identify the issues that require to be addressed. The study then broadens the research scope on environmental management by offering the antecedents and consequences of green mindfulness. A better understanding of the mechanisms supporting green mindfulness advances selection and development efforts aimed at improving environmental performance. The following demonstrates a review of literature on mindfulness concept in organizational behavior, illustrates an explanation for the association between the mindfulness concept and environmental management, and addresses a discussion on the potential antecedents and consequences of green mindfulness.

## 2. Mindfulness Concept in Organizational Behavior

Mindfulness denotes a psychological status that reflects on individuals’ cognitive qualities [36,37]. It is a state of awareness which illustrates dynamic information processing, design and refinement of diverse categories, as well as the consciousness of multiple perspectives. At the individual level, mindfulness can be conceptualized as a cognitive ability that is reflected by openness to novelty, alertness to distinction, awareness of multiple perspectives, sensitivity to different contexts, and orientation in the present [34,35,38,39]. Openness to novelty includes the ability to think about new kinds of stimuli. Alertness to distinction includes the ability to compare and make judgments about the differences and similarities. Awareness of multiple perspectives includes the ability to view things from different points of view. Sensitivity to different contexts includes the ability to detect the characteristics and changes of particular situations. Orientation in the present includes the ability to pay attention to the immediate situation [39].

Individual mindfulness focuses on the ability to continuously create and use new categories in perception and interpretation of the world [28,38]. Mindfulness captures a quality of consciousness characterized by clarity and vividness of current experience and functioning. In contrast, mindlessness is characterized by less conscious states, where people tend to function habitually and automatically [40]. People who are engaging in a task mindfully are able to make more relevant distinctions about phenomena in their environments, explore a wider variety of perspectives, and enables them to adapt to changes in those situations [24,41].

Although mindfulness is initially defined as an individual-level characteristic, the concept of mindfulness has been subsequently extended to the organization level [42]. However, a mindful organization is not just the sum of mindful individuals [43]. At the organization level, mindfulness is defined as an organizational capability for a firm to operate under the conditions that are considered by high functional risks and technological complexity, and with slight scope by trial and error. Firms make mindful decisions based on reasoning grounded in their own organizational facts and specifics [26]. In recent years, the mindfulness concept has been introduced into organizational studies via the examination of individual and collective mindfulness within an organization.

In a study of high reliability organizations (such as emergency departments in hospitals, air traffic control systems, nuclear power generating plants, etc.), Weick and his colleagues [27,44] indicated five mindfulness concepts to avoid accidents and failures as well as to sustain under complex and high risk situations within the organizations. These five concepts regarding concern with failure, reluctance to abridge interpretations, sensitivity to procedures, commitment to resilience, and obedience to expertise, have been considered the gauges of mindfulness in organizations in conducting daily operations to benefit organizational mindfulness [25,27].

Concern with failure assumes that the errors, problems, and unusual occasions, no matter how insignificant, are possibly vital indicators of potential problems that should be considered to sustain an organization to face unexpected situations. This implies that each occasion should be attended to in order to recognize its causes and effects. Mindful organizations regularly examine failures, regardless of seriousness, as signs to a huge problem. These organizations treat any irregularity, or slight disruption, as a symptom that may result in severe outcomes. Mindful organizations consider any unexpected changes as if something goes wrong and might be catastrophic. They are suspicious of potential liabilities related to ongoing success, and are particularly concerned with contentment, temptations to decrease the safety margins, and automatic processing. Mindful organizations encourage individuals who recognize mistakes, then analyze them as to make enhancements to prevent future occurrences from failures [27].

Reluctance to abridge interpretations means taking distinct steps to stop making things easy or oversimplifying the daily operations. Members in mindful organizations share the conception that the surroundings are either complicated or unpredictable so that expectations are questioned rather than presupposed. Mindful organizations identify challenges and assumptions all the time, and remind themselves by seeing as much as possible, regardless of how complex, unstable, and unpredictable these may be. They push the boundaries for acquiring knowledge without destroying the nuances that diverse people identify. Mindful organizations do not restrain or limit the viewpoints within the organizations. Employees are recruited and promoted from different backgrounds, and are also encouraged to be skeptical and critical. Therefore, employees are told to pay close attention to their jobs and duties, with the belief of preventing oversimplification [27].

Sensitivity to procedures denotes to having detailed understanding of every aspect of the business either operationally or strategically. Mindful organizations are conscious of operation procedures and devote themselves to understanding situational awareness. This capability enables them to adjust and to avoid potential mistakes. Therefore, organizations with procedural and operational sensitivity could thoroughly evaluate routine tests to uncover possible or occurring failures that might become an unexpected disaster. Mindful organizations regard near misses as evidence of successes, and develop the environment in which the reporting of defects and faults are stimulated. The purpose is to ensure continuous communications within the organization levels, while clearly defining and valuing each individual level. Mindful organizations are constantly looking for ambiguity or problems which may result in failures within their workflows and processes. Individuals are thus encouraged to find mistakes and to question the current assumptions [27].

Commitment to resilience is the notion to be mindful of mistakes and correct the errors before becoming worse, which also means to anticipate issues with possible resolutions before the problem occurs. Resilience means the capability to bounce back rapidly when encountering disasters. Mindful organizations develop the capacity to extemporize and recover from setbacks. These organizations resist the inducement to agonize forevermore about what has occurred and instead act to improve. They are willing to act swiftly, to analyze the situation, to engage in learning, and then to act and respond quickly [27]. Obedience to expertise implies seeking the qualified individuals for decision-making or job completion. The mindful organizations with hierarchal structures not only reveal how decisions are made but have the ability to distinguish from low, medium, and high volume times to intensify urgent situations properly by the individual with the most expertise. Expertise is not necessarily matching with the hierarchical position but considering the capabilities to make the adequate decisions, and also conveying and executing the decisions as much as possible to the lowest levels of the organizations [27].

In summary, mindfulness of the organizations stands for the individuals’ and organizations’ capabilities to attain reliable outcome performance in a changing environment which depends on how individuals and organizations reflect, perceive the surroundings and collect information, and on whether they are able to alter their perspectives to reveal the conditions. Mindfulness entails the need to enhance situational awareness on an ongoing foundation, to cast suspicion, and to examine further to solve doubtfulness. Though business operations are usually performed by companies under significantly less rigorous conditions than those with high reliability, adopting the five characteristics above in organizational operations could lessen the opportunities of failure by avoiding mistakes at the beginning [27]. According to the resource-based view, mindfulness can be regarded as an important resource to reinforce competitive advantage [13,14]. Therefore, mindfulness can be regarded as a desirable asset or state that all businesses, regardless of their line of operation, may strive to achieve. Further, mindfulness will make an organization more skilled in managing unexpected conditions [17,31].

As a result, since Weick and his colleagues’ studies focused on high reliability organizations [27,42,44,45], the concept of mindfulness has been receiving heightened interests in the context of different aspects of organizational behavior such as organizational learning and attention [16,46,47,48], entrepreneurship behavior [49], organizational information technology innovation [22,23,24,39,48,49,50,51,52], organizational task performance [27,53], organizational dynamic capabilities [54,55,56], organizational accident management [57], organizational implementation of complex health improvement programs [58,59], quality management [32,60], school management [31,35,43,61,62], human resource management [63], marketing strategy implementation [33,34], product failure management [64], and RFID implementation [65,66]. While mindfulness is commonly considered to be a favorable property or characteristic to possess for a firm, there is still a lack of study regarding applying the concept of mindfulness to environmental management [15,29].

## 3. Application of the Mindfulness Concept to Environmental Management

We argue that the notion of mindfulness is also proper for environmental management. According to the previous discussions, this study holds that mindfulness in environmental management, here denoted as *green mindfulness*, is a way of working during environmental management marked by the willingness to consider alternative perspectives, focus on the present, give attention to operational detail, and have an interest in exploring and understanding failures [27,36]. Firms need to keep mindfulness thinking when engaging in environmental management.

To improve environmental performance, many firms engage in environmental management activities reactively or proactively [67,68]. Many researchers have used a variety of theories to analyze various environmental management issues. Among an amount of environmental management studies, however, little attention has been paid on analyzing mindful behaviors associated with environmental management implementation within organizations [6]. Applying environmental perspectives into the business operations requires exploring the combinations of new resources as well as using existing resources in new ways [18,19]. Executing environmental management usually encompasses adopting modified or new techniques and processes that may reduce environmental damages. The implementation of these new green concepts or practices could not guarantee that there is a pervasive usage of the new concepts or practices within the firm to fulfill the environmental benefits [7,69]. No matter if undertaking environmental management reactively or proactively, green concepts or practices can be introduced with an abundant passion; yet, these may not be thoroughly used among corporations [3,5].

Corporate greening usually begins with the awareness of top management for the requirement of corporate responses to environmental issues, leads to policy commitment, and ideally, ends with implementation at the operational level [7]. Environmental management systems are frequently subject to significant hype [20,70]. There are essential difficulties that are associated with choosing suitable environmental green concepts and practices from a number of possibilities. As part of this, environmental green practices may be shown in ways that amplify the applicability scope, overemphasize advantages, underestimate challenges, and pursue to create the emergency by appealing that a widespread adoption in industry-level is expected and that organizational implementation is absolutely critical for continuous corporate success and sustainability [71,72]. Therefore, the bandwagon effects or social pressures may influence a company’s decisions significantly regarding environmental management [67,73]. Additionally, the high uncertainties, worries, hype, as well as “me too” motivations may result in engaging in environmental management activities in ways that are only partly grounded in the facts and specifics of the organizations [26,74]. Consequently, pursuing to carry out environmental management might encounter the daunting duty of connecting the gaps between deployed technologies, organizational capabilities, and business demands for the organization [8,18].

Engaging in environmental management usually calls for substantial resources regarding investment of an organization. Environmental management sometimes constitutes a decision-making scenario with intricate information processing which involves making sense of the green concepts and practices that an organization is not acquainted with, and is usually characterized by uncertainties and ambiguities over the ramifications of the execution processes [7,8,9]. Hence, supervisors are confronted with the responsibility of analyzing the consequences of environmental management on their company. Under such conditions, deciding on whether a green concept or practice is beneficial for the organization, whether the timing of the operation is suitable, and how the execution is best performed, all requires firms to be mindful of environmental management with reasoning grounded in the realities of the company. As a result, mindfulness thinking has become important for environmental management. Firms need to remain in mindful thinking when engaging in environmental management [10,35]. Mindfulness is therefore a characteristic that is believed to aid in making contextually differentiated clarifications of situations and information scenarios in organizational decision-making [6,16,17,43].

The mindfulness approach is also suitable for environmental management. Adopting the criteria of environmental management into the operations of the firm requires exploring the combinations and the deploying of new and existing resources together in innovative ways. Conducting environmental management might involve using new or modified techniques or procedures to reduce the environmental burden, which can also be considered the process of organizational innovation [20,21,22]. The notion of mindfulness has been widely used to examine the engagement with innovations of the organizations [24,25,26,52,54]. The implementations of environmental management are normally characterized by new and complex technical knowledge and process changes, resulting in unexpected or uncertain outcomes. When implementing an innovation, mindfulness pertains to attend to the innovation with a contextually differentiated reasoning based on the firm’s own facts and specifics [26]. Accordingly, mindfulness in environmental management not only refers to being experienced about the green concepts, green practices, and the implications, but also being able to contextualize the knowledge regarding the concepts or practices which are based on the particular situations prevailing in the firm and the implications on the operation. Mindfulness might have implications in environmental management, as the decision of evaluating and adopting green concepts or practices underlines a company’s endeavor to make sense of something that is uncertain and may bring about unexpected consequences.

In a study of high reliability organizations, Weick and Sutcliffe [27] argued that the approach of mindfulness reveals the point that many catastrophes are caused by small errors instead of the occurrence of large or disastrous ones. Similarly, the successful environmental management is usually the result of the outgrowth of an incidental combination of numbers of small ones rather than a single large decision or plan [8,73]. Minor disruptions, mistakes, or chances are most likely to be observed on the front lines of the company where individuals who participate in the daily operations reside. If these unpredicted conditions are handled promptly, there is a chance to avoid the acceleration into large problems or to control them to enable change. Mindful organizations encourage people to report occurring or potential mistakes, near misses, improvement opportunities, and to treat these mistakes and misses as systemic matters rather than individual occasions. With regard to the environmental management, mindful organizations who are concerned with failure, sensitivity to operations, and obedience to expertise may pay more attention to the possible drawbacks associated with applying the concepts or practices of a green environment. In addition, mindful organizations are more likely to empower their experienced team members and allow them to deal with an emergent problem as well as to act on incipient opportunities. It is more likely to recognize problems that must be handled to maintain current procedures, but also important to realize problems as indicative of the system issues which offer opportunities for advance execution of environmental management [75]. As a result, all these aspects of organizational mindfulness can prepare a company to be better able to manage both the preliminary introduction and subsequent execution of the environmental management.

For companies pursuing to undertake environmental management with their maximized capabilities, reluctance to abridge interpretations is important because the process of the environmental management is prone to be surprised, complex, and unexpected. The identification and evaluation of green concepts or practices requires companies to engage in an environment with hype-saturated and noisy information. The green concepts or practices are measured regarding their impact on other companies or in the contexts based on second-hand information [76,77]. In this condition, there is the inducement to use the logic of bandwagon and assume other companies’ successes in environmental management as a robust indicator of what a company can expect when executing environmental management [67,78]. Reluctance to abridge interpretations and commitment to resilience can assist and protect companies against this, keeping the companies concentrated on the need to know how a green concept or practice can fit specifically with the unique characteristics and particular needs of their organizations [24]. Similarly, mindful firms with both reluctances to abridge interpretations and commitment to resilience are more likely to identify hidden opportunities for implementing environmental management because they are less likely to assume that the current processes and structures are necessarily the most appropriate. Mindful companies are likely to be able to steer the challenges of implementing environmental management because these companies are less likely to become hooked on preliminary claims and plans, and also the first round of deployment objectives.

Levinthal and Rerup [46] addressed that sustained mindfulness in a firm requires the capacity to respond to unanticipated signals from one’s situation as well as the attentiveness to one’s situation. This mindfulness concept encompasses cognitive and behavioral dimensions, and provides a perspective for studying organizational mindfulness. The cognitive aspect of mindfulness refers to collecting relevant information of both internal and external environments, and being able to realize what the information means. It connotes the awareness of multiple perspectives of the present experience and reality [38,40]. The behavioral aspect refers to the enactment of the processed information, such as routines that provide repertoires of action [46] and motivation that causes the arousal to act [79,80]. Therefore, mindfulness denotes a situation of being attentive and conscious. It is a desirable organizational property that is primarily in the context of managing day-to-day operations of organizations [42]. It considers not only how an organization creates value by integrating its competencies but also how it acts to attain business performance, i.e., monitors its internal and external needs [51]. Accordingly, firms also require mindfulness in implementing environmental management. Mindfulness approach, characterized by an ability to detect changes in the environment and to contextually interpret their importance for the firm, is argued to be important for environmental management. Companies are required to retain green mindfulness when engaging in environmental management.

Drawing on the literature of mindfulness, we define green mindfulness as a way of working during environmental management marked by the willingness to consider alternative perspectives, focus on the present, give attention to operational detail, and have an interest in exploring and understanding failures [6,27,36]. Green mindfulness can also be discussed at the individual and organizational levels. At the individual level, green mindfulness can be conceptualized as a cognitive green ability that is reflected by alertness to distinction, awareness of multiple perspectives, openness to novelty, sensitivity to different contexts, and orientation in the present [28,38,39]. At the organizational level, green mindfulness can be conceptualized as a cognitive green ability that is reflected by reluctance to simplify interpretations, preoccupation with failure, commitment to resilience, sensitivity to operations, and deference to expertise [27].

## 4. Conceptual Model Development

In addition to investigating the nature of green mindfulness in firms, understanding the antecedents and consequences of green mindfulness can help firms appreciate the potential drivers and barriers to implementing environmental management. Although many studies in the literature have explored the antecedents and consequences of environmental management [67,70,81,82,83], there is still a lack of research on the antecedents and consequences of mindfulness [6,35]. Prior research on organizational mindfulness issues has focused on demonstrating its contributions to organizational decision-making and performance [15,35]. To fill the research gap, this paper attempts to propose a conceptual model describing the antecedents and consequences of green mindfulness based on a review of literature related to environmental management.

Many researchers have used a variety of theories, including institutional theory, resource-based view, stakeholder theory, innovation diffusion theory and others, to propose various explanations for firms’ implementation of environmental management. Among these theories, the resource-based view has been widely used in the literature [8,16,82,83,84,85,86,87,88]. Furthermore, according to the resource-based view, mindfulness can be regarded as an important resource to reinforce competitive advantage [13,14]. Mindfulness can be regarded as a desirable asset or state that all businesses, regardless of their line of operation, may strive to achieve. It will make an organization more skilled in managing unexpected conditions [17,31]. Therefore, this study will develop the conceptual model from the perspective of a resource-based view.

An extensive literature review on environmental management implementation reveals that antecedents and consequences of environmental management can be analyzed based on individual and organizational levels in the study [67,70,81,82,83]. Many studies have discussed the influences of a variety of organizational factors on environmental strategy [67,82]. Implementing environmental management also requires a great deal of individual effort and persistence [20,28,70]. Drawing on the resource-based view, the main antecedents and outcomes of environmental management include organizational capabilities, external stakeholders’ pressures, firm size, and quality of human resources. The main outcomes are competitive advantage and firm performance, particularly economic performance and environmental performance [8,84,85,86]. The resource-based view suggests that organizational strategy, organizational capability, and human capital are primary factors influencing the utilization of firm resources to achieve competitive advantage [87,88,89,90,91,92]. The organizational strategy and capability include external stakeholder focus [88], organizational learning capability [93], organizational culture [94], business strategic proactivity [95], firm size, and others [87,88,89,90]. The human capital includes employees’ activity role [96], self-efficacy [97], learning attitude [98], and others [89,90,91,92].

As a result, this study will take external stakeholder focus, organizational learning capability, organizational ethical culture, business strategic proactivity, and firm size as the organizational antecedents, and take green activity role, green self-efficacy, and learning attitude of employees as the individual antecedents in the conceptual model, as these variables have also been commonly studied in the literature [67,70,81,82,83,84,85,86,87]. Regarding the consequences of green mindfulness, this study includes firms’ environmental performance and economic performance, and individual role overload in the proposed model because firms’ performance have been widely studied in the literature of mindfulness [13,33] and environmental management [67,81,83], and role overload has been assumed to be an important individual consequence of mindfulness [38,39].

**Figure 1:**
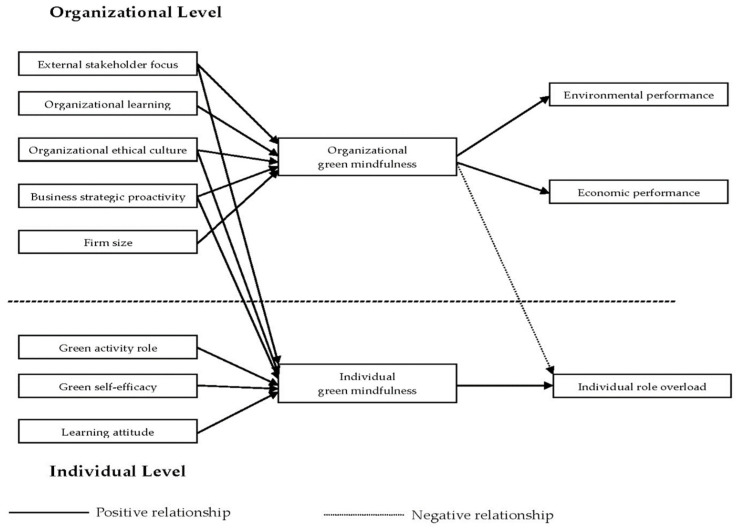
Conceptual Model.

Recognizing the mindfulness phenomena being inherently multilevel in nature [6,35], analyzing the antecedents and consequences of green mindfulness should be from a multilevel perspective. Therefore, a multilevel conceptual model of green mindfulness, as shown in Figure 1, is proposed in this study. Both antecedents and consequences of green mindfulness are analyzed based on individual and organizational levels in the study. The associations among those variables in the conceptual model will be discussed in the following sections.

## 5. Antecedents of Green Mindfulness

According to the proposed conceptual model, the antecedents of green mindfulness can be divided into organizational-level and individual-level predictors which motivate organizational and individual green mindfulness within a firm.

### 5.1. Organizational Antecedents of Green Mindfulness

There are five organizational antecedents in the proposed conceptual model, including external stakeholder focus, organizational learning capability, organizational ethical culture, business strategic proactivity, and firm. The following discusses the influences of these organizational antecedents on organizational and individual green mindfulness.

#### 5.1.1. External Stakeholder Focus

Stakeholders are groups or individuals who can exert influences on a company’s activities and are also influenced by the company’s activities. External stakeholder focus implies the degree that companies incorporate external stakeholders into their environmental management and business strategy [99,100]. Stakeholder pressure has been considered as the most prominent factor influencing a company’s environmental strategy [82,83]. According to the resource-based view and stakeholder theory, companies implement activities to satisfy their primary stakeholders. Environmental consciousness of a firm implies harmonizing green performance with stakeholders’ expectations. Under the situations of high stakeholder pressure, companies may be apt to be reluctant to simplify stakeholders’ various environmental requirements, and to keep commitment to resilience. Furthermore, reflecting company employees’ collective perceptions about the relevance and value of environmental management as a critical firm function, external stakeholder focus is identified as important for companies that are highly interdependent with external constituents [4,101]. Companies that collectively value environmental management practices should be more likely to incorporate external stakeholders into business strategy, monitor these practices accordingly, and actively encourage green mindfulness throughout the company’s operations. Therefore, the following proposition is proposed:

**Proposition** **1:** *A firm’s focus on external stakeholders will have a positive effect on individual and organizational green mindfulness*.

#### 5.1.2. Organizational Learning Capability

Organizational learning capability is considered as the organizational characteristics that facilitate the organizational learning process towards environmental management. Environmental management practices incorporate both tacit and explicit knowledge [93]. The tacit knowledge may be inherent in identifying sources of pollution, reacting quickly to accidental spills, and proposing preventive solutions [68,98]. A green concept or practice containing a lot of tacit knowledge needs laborious efforts to learn. The difficulty in learning and sharing tacit knowledge makes it relatively difficult to implement a green concept or practice, and consequently, the company may be likely to be reluctant to simplify interpretations and deference to expertise. Furthermore, because several environmental management practices are additions to firms’ current technologies and processes, implementing environmental management is not a single event but can be described as a process of knowledge accumulation and integration. Managers need to explore new knowledge for making decisions, deploying resource combinations, and performing tasks while managing the uncertainties and ambiguities of societal expectations around environmental issues, such as new technologies, regulations, and corporate environmental impacts [100]. Organizational learning is central to implement environmental management that requires companies to gain knowledge of new ways of doing works [93]. The firm members’ environmental training, such as self-learning, professional education, and job training, is, to a certain extent, a determinant factor of the level of development of the firm’s environmental strategy. Enhanced firm members’ awareness of environmental issues leads to improved individual environmental behavior [83]. Therefore, the following proposition is proposed:

**Proposition** **2:** *A firm’s organizational learning capabilities will have a positive effect on individual and organizational green mindfulness*.

#### 5.1.3. Organizational Ethical Culture

Organizational ethical culture denotes a shared understanding of the ethicality by the members in the organization. Several studies argued that organizational culture would govern how rewards systems are defined within the organization, and the ways in which an organization deals with mishaps and failures [94]. Organizational culture is also associated with organizational mindfulness behavior. According to a study on high reliability organizations, Weick and Sutcliffe [27] argued that informed organizational culture can foster mindfulness among organizations. Regarding environmental management, a firm’s environmental strategy is related to organizational ethical culture [102]. Environmental policy making and auditing activities must be implemented in an ethical corporate culture [103]. Organizational ethical culture is an interplay of formal and informal corporate systems that can support ethical organizational behavior in the firms. Formal ethical systems include authority structures, organizational policies, and reward systems. Informal systems include perceived organizational expectations and norms, and peer behavior [104]. Employees’ perceptions of the ethical culture may affect the likelihood of their unethical or dysfunctional behaviors as well as their affective outcomes such as job satisfaction and organizational commitment. The perceived ethical culture essentially defines what is regarded as acceptable or legitimate in the firm [105,106]. As implementing environmental management can be regarded as ethical behavior [107,108], the following proposition is proposed:

**Proposition** **3:** *A firm’s organizational ethical culture will have a positive effect on individual and organizational green mindfulness*.

#### 5.1.4. Business Strategic Proactivity

Business strategic proactivity is regarded as the ability to develop administrative, entrepreneurial, and engineering skills and processes to actively seize new opportunities rather than merely react to changes [95]. A firm’s business strategic attitude is relevant for the selection of environmental strategies [19,68,82]. Environmental advancements are associated with a firm’s strategic proactivity. This capability includes increasing innovation in managing strategic issues, adopting organizational processes and structures to facilitate innovation and reduce uncertainty, implementing flexible technologies to facilitate a speedy response, and identifying new opportunities for technological development [95]. The development of a proactive environmental strategy is positively related to the capability of business strategic proactivity [109]. A firm engaging in environmental management proactively would be willing to explore new environmental management practices and provide more resources required for the implementation of green practices. An environmental-proactive firm would be mindful in environmental management. Therefore, the following proposition is proposed:

**Proposition** **4:** *A firm’s business strategic proactivity will have a positive effect on individual and organizational green mindfulness*.

#### 5.1.5. Firm Size

Firm size, which can be measured by total assets, total sales, or number of employees, is widely taken as a relevant organizational factor influencing firms’ environmental activities [67,82]. In general, large firms tend to pay more efforts in implementing environmental management than small ones. According to the resource-based view, the arguments used to explain this effect focus on different aspects: (1) the environmental efforts of large firms have significant and positive impacts on a larger number of stakeholders; (2) large firms receive more pressure from the economic and social environments and institutions; (2) they have more resources to devote to environmental management; (3) their scales allow them to face the indivisibilities associated with green management, that is, those required investments in technology, human resources or certifications [82]. Therefore, the following proposition is proposed:

**Proposition** **5:** *There is a positive association between firm size and individual, and organizational green mindfulness*.

### 5.2. Individual Antecedents of Green Mindfulness

There are three individual antecedents in the proposed conceptual model, including green activity role, green self-efficacy, and learning attitude of firm members. The following discusses the influences of these individual antecedents on individual green mindfulness.

#### 5.2.1. Green Activity Role

According to the role theory [96], the green activity role denotes an individual perception of his or her responsibilities in environmental management implementation. Green activity roles in a company are expected to provide a structure that directs employee’s efforts and reinforces employees’ environmental management responsibilities [96]. Employee roles are generally conceptualized as socially constructed frameworks of behaviors that are expected and appropriate [110,111]. Utilizing environmental criteria in company operations sometimes requires deploying existing resources in new ways and exploring new resource combinations. Undertaking environmental management usually involves using new or modified processes and techniques in addition to the firm members’ current tasks. As environmental management activities are sometimes complicated and can directly compete with other activities and processes for time and attention, employees are unlikely to engage in these additional activities unless they believe these activities to be a part of their job role responsibilities [96,101]. According to the role theory, organizational members develop expectations about their own roles that direct beliefs about appropriate role behaviors [111]. As a result, firm members would sometimes provide indications about rewards and sanctions associated with role compliance in an effort to affect role conformity and facilitate the accomplishment of firm objectives [81]. Green activity roles may experience internal pressures to gain social approval and conform to expectations. They may successfully demonstrate valued role performance and intrinsic satisfaction from fulfilling challenging roles when undertaking environmental management. Therefore, the following proposition is proposed:

**Proposition** **6:** *A firm member’s perception of his or her green activity role will have a positive effect on individual green mindfulness*.

#### 5.2.2. Green Self-Efficacy

According to the social cognitive theory [97], green self-efficacy in this study denotes as the conviction that a firm member can successfully execute their behavior to produce the outcomes of environmental activities. Green self-efficacy should provide firm members with the confidence in their own capabilities to successfully implement environmental management. It implies an employee’s confidence in his/her ability to successfully manage relationships with important parties external to the company. As environmental management activities are sometimes complicated and require a great deal of persistence and effort, employees need to feel confident that they can perform such challenging environmental management activities as well as their other job responsibilities. An employee’s green self-efficacy should be important in implementing environmental management. Highly efficacious employees will feel confident while interacting with external stakeholders and believe that they have the abilities to effectively manage various external impacts on the company. Due to their confidence, these employees are more likely to perceive the challenges and demands associated with environmental management as opportunities to excel rather than obstacles to avoid. They are not only more apt to take on environmental management, but also are apt to control the negative emotional reactions that often arise during challenging situations [97]. Consequently, organizational members with greater self-efficacy will set higher personal goals regarding environmental management and will be more likely to generate appropriate methods for implementing these challenging behaviors successfully [111]. Therefore, the following proposition is proposed:

**Proposition** **7:** *A firm member’s green self-efficacy will have a positive effect on individual green mindfulness*.

#### 5.2.3. Learning Attitude

Learning attitude in this study refers to the responses and subsequent acts of a firm member toward learning new knowledge [83,98]. Implementing environmental management is a complex process requiring cross-disciplinary coordination and significant changes in the existing operation process [87]. It is intensive in human resources and depends on the development and training of tacit skills through the firm members’ involvement [18,83]. Mindfulness requires reluctance to simplify interpretations and an awareness of sensitivity to operations [27]. In the context of environmental management, this implies the consideration towards the different aspects of the green practice on the company’s operational advantages. Qualified employees with the ability to consider various methods to solve a problem and elaborate on the details of an idea will make a company aware of the multiple aspects of environmental management. To equip firm members with environmental awareness and knowledge, firms should provide environmental training for their members. According to organizational learning theory, learning effect and learning efficiency would be influenced by an individual’s learning attitude. A firm member with a higher learning attitude toward new knowledge will be fond of learning environmental knowledge via environmental training and possess higher environmental awareness [98]. Therefore, the following proposition is proposed:

**Proposition** **8:** *A firm member’s learning attitude will have a positive effect on individual green mindfulness*.

## 6. Consequences of Green Mindfulness

According to the proposed conceptual model, the consequences of green mindfulness can be divided into organizational-level and individual-level categories. Organizational consequences consist of a firm’s environmental performance and economic performance. Individual role overload is assumed to be the individual consequence of green mindfulness.

### 6.1. Organizational Consequences of Green Mindfulness

Environmental performance refers to the effects of business activities and products on the natural environment; for example, the degrees of preventing pollution impact and reducing resource consumption [67,82]. Economic performance refers to how well a firm can use assets from its business activities and generate revenues; for example, return on equity, return on capital employed, and gross profit to sales ratios [72,112]. This study expects organizational green mindfulness to be positively related to firms’ environmental and economic performance. Existing studies of organizational mindfulness and environmental management support this assertion. It is believed that better environmental performance can be achieved when environmental aspects are systematically identified and managed. The main goal of implementing environmental management practices is to improve environmental performance. Anecdotal information and many studies have provided a body of evidence for the positive environmental benefits from implementing environmental management [67,82]. Additionally, there are many studies supporting the hypothesis that implementing environmental management is positively related to economic performance [72,112]. Furthermore, organizational mindfulness can help firms pay much attention to the utilization of management practices and concepts [17,27]. Therefore, the following proposition is proposed:

**Proposition** **9:** *Organizational green mindfulness will have positive effects on a firm’s environmental performance and economic performance*.

### 6.2. Individual Consequences of Green Mindfulness

Role overload is a type of person role conflict in which he or she must decide on which activities to do and which to delay [111]. It exists when an employee has too much work to do at the same time. In this study, we suppose that individual role overload which a firm member experiences is an individual consequence of green mindfulness. Despite the potential benefits of green mindfulness for companies, keeping green mindfulness requires considerable efforts, and is challenging and stressful. These characteristics are likely to lead to role overload [38,39]. The costs in terms of additional efforts are largely attributable to the fact that those employees engaging in green mindfulness are responsible for actively managing various jobs either sequentially or simultaneously. Implementing these additional behaviors requires the employees to be connected both internally and externally to their firms which can contribute to role overload. Furthermore, because green mindfulness requires a balance with internal processes [38,39], employees may find that they have greater role demands when they engage in additional environmental management activities. This may lead to role overload because of the increasing number of role demands an employee experiences. Therefore, the following proposition is proposed:

**Proposition** **10:** *Individual green mindfulness will have a positive effect on the amount of individual role overload that firm members experience*.

Although firm members will experience greater role overload when they have high green mindfulness, it is expected that individual role overload may be reduced when the company as a whole engages in organizational green mindfulness [101,111]. Companies engaging in higher organizational mindfulness may receive more supports and resources from top managers, which may help employees manage their environmental responsibilities and obligations, and help minimize employees’ role overload [27,83]. Organizational green mindfulness may reduce the degree of role overload to which employees experience. Therefore, the following proposition is proposed:

**Proposition** **11:** *Organizational green mindfulness will have a negative effect on the amount of individual role overload that firm members experience*.

## 7. Summary

Environmental management has received sustained research interest over time due to the multitude of factors that can affect environmental efforts. While many researchers have used a variety of theories, including institutional theory, resource-based theory, stakeholder theory, organizational learning theory and others, to propose various explanations for firms’ implementation of environmental management, there is still a lack of research focusing on utilizing the mindfulness concept in environmental management. This study attempted to apply the concept of mindfulness to environmental management and proposed a multilevel conceptual model describing antecedents and consequences of green mindfulness.

Drawing on the literature of mindfulness, this study defined green mindfulness as a way of working during environmental management marked by the willingness to consider alternative perspectives, focus on the present, give attention to operational detail, and have an interest in exploring and understanding failures. Green mindfulness can also be discussed at the individual and organizational levels. At the individual level, green mindfulness can be conceptualized as a cognitive green ability that is reflected by alertness to distinction, awareness of multiple perspectives, openness to novelty, sensitivity to different contexts, and orientation in the present. At the organizational level, green mindfulness can be conceptualized as a cognitive green ability that is reflected by reluctance to simplify interpretations, preoccupation with failure, commitment to resilience, sensitivity to operations, and deference to expertise.

In addition, this study proposed a multilevel conceptual framework describing the influences of organizational and individual antecedents on green mindfulness, and the organizational and individual consequences of green mindfulness, as illustrated in Figure 1. The proposed model assumed that both individual and organizational green mindfulness are positively related to five organizational antecedents, including external stakeholder focus, organizational learning capability, organizational ethical culture, business strategic proactivity, and firm size. Individual green mindfulness is positively related to three individual antecedents, including a firm member’s perception of his or her green activity role, the firm member’s green self-efficacy, and learning attitude. Organizational green mindfulness has positive effects on a firm’s environmental performance and economic performance. Individual green mindfulness has a positive effect on individual role overload that firm members experience; however, organizational green mindfulness may have a negative effect on the individual role overload of a firm member.

Undertaking environmental management is generally believed to impart strategic and competitive benefits to the firms. However, it also involves significant resource commitments on behalf of the firm. Chances of failing to successfully implement environmental management are often quite high. Thus, firms are usually faced with complex situations of deciding to implement a green concept or practice that is relatively new to the company and uncertain in expected outcomes. Environmental management may be starting with a great enthusiasm; nevertheless, it may fail to be thoroughly deployed among many firms. Based on the literature review and theoretical considerations, we think it is time to add more understanding on green mindfulness in environmental management. Mindfulness approach, characterized by an ability to detect changes in the environment and to contextually interpret their importance for the firm, is argued to be important for environmental management. Companies are required to retain green mindfulness when engaging in environmental management.

Although there is still not an abundance of research on mindfulness in organizational behavior, the studies that deal with mindfulness provide some useful clues to the present study. While there is an increase in theoretical and case-based empirical works regarding the nature of mindfulness, understanding the antecedents and consequences of mindfulness remains a challenge. The present study can broaden the scope of research both on environmental management and mindfulness theory. Based on the proposed green mindfulness concept, the future study can equip research on green mindfulness with some empirical evidence. In addition, future study can also extend the proposed model by including other organizational and individual antecedents and consequences in the analysis. A comparison between mindfulness and other conceptualizations of thinking modes, or reconsidering mindfulness as a domain state measured by frequency and comprised of proposed antecedents, can also be conducted in future studies.

## Data Availability

Not applicable.
